# *Capnocytophaga* Keratitis: Clinical Presentation and Use of Metagenomic Deep Sequencing for Diagnosis

**DOI:** 10.1097/ICO.0000000000001790

**Published:** 2018-10-19

**Authors:** Gerami D. Seitzman, Praneetha Thulasi, Armin Hinterwirth, Cindi Chen, Jessica Shantha, Thuy Doan

**Affiliations:** * Francis I. Proctor Foundation, University of California San Francisco, San Francisco, CA;; † Department of Ophthalmology, University of California, San Francisco, CA; and; ‡ Department of Ophthalmology, Emory University, Atlanta, GA.

**Keywords:** *Capnocytophaga* keratitis, infectious keratitis, metagenomic deep sequencing

## Abstract

**Purpose:**

To report our experience with 2 cases of *Capnocytophaga* keratitis.

**Methods:**

This is a retrospective study of case reports. We present the clinical presentation, diagnosis, and treatment strategies of 2 patients who presented with *Capnocytophaga* keratitis.

**Results:**

Both patients had risk factors including systemic immune compromise and ocular trauma. Both patients had robust inflammatory keratitis with necrosis. Case 1 demonstrates identification of *Capnocytophaga* with traditional microbiologic techniques. Case 2 demonstrates the use of unbiased metagenomic deep sequencing for identification of this unusual corneal pathogen.

**Conclusions:**

*Capnocytophaga* is a rare and aggressive infection. Even when traditional culture identifies the pathogen rapidly, keratitis can progress to perforation. In cases of severe keratitis in which traditional culture methods are unrevealing, metagenomic deep sequencing has potential to provide actionable diagnoses.

*Capnocytophaga* keratitis is a rare and aggressive ocular infection.^1–4^ The largest case series, including 10 cases, was reported in 2000.^1^ Five of these cases resulted in enucleation. We report our recent experience with 2 cases of *Capnocytophaga* keratitis to remind clinicians of this unusual and destructive infection and to highlight how both diagnosis and management of this infrequent bacterial infection are often challenging.

## CASE REPORTS

### Case 1

**FIGURE 1.**
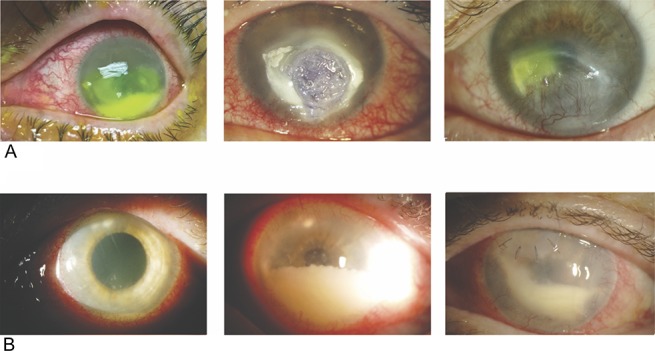
Slit-lamp photographs of both cases. A, Case 1; left panel: presentation photograph with infiltrates and hypopyon; middle panel: 4 days after presentation with perforation requiring glue; right panel: 2 months after glue. B, Case 2; left panel: presentation photograph with superior mid-stromal peripheral and paracentral subepithelial infiltrates with hypopyon; middle panel: progressive inflammation; right panel: small superonasal patch graft was performed after diagnostic deep corneal biopsy. Robust AC inflammation remains. Fluid from an AC tap at this point was sent for metagenomic deep sequencing.

A 58-year-old man presented with a medical history significant for chronic myelogenous leukemia (CML) status after allogenic bone marrow transplant. Ocular history was significant for severe ocular graft-versus-host disease, keratoconjunctivitis sicca, and bilateral neurotrophic keratopathy. His left eye was recently treated for a culture-positive *Streptococcus viridans* corneal ulcer with hypopyon, and he recovered 20/60 acuity. The patient re-presented (Fig. 1A, left) with new, left large central corneal epithelial ulceration, 2 paracentral areas of corneal infiltration, and a 3.5-mm hypopyon. Hourly fortified cefazolin (50 mg/mL) and topical moxifloxacin were initiated. Three days after culture, microbiology identified growth of numerous *Capnoctyophaga cynodegmi* species. The patient reported, while celebrating his recovery from *S. viridans* keratitis, that he let his dog lick him all over his face, including his neurotrophic corneas. Four days after presentation, the patient developed Seidel-positive inferior paracentral perforation requiring an emergency glue procedure (Fig. 1A, middle). Because sensitivities for this rare pathogen require send-out evaluation, a review of previous *Capnocytophaga* case reports^1–3^ suggested treatment with topical clindamycin. Compounded clindamycin 5% was initiated hourly. The glue remained in place for 2 months and subsequently fell off. Visual acuity improved to 20/200. The globe remained intact, and the area of previous perforation had vascularized (Fig. 1A, right).

### Case 2

**FIGURE 2.**
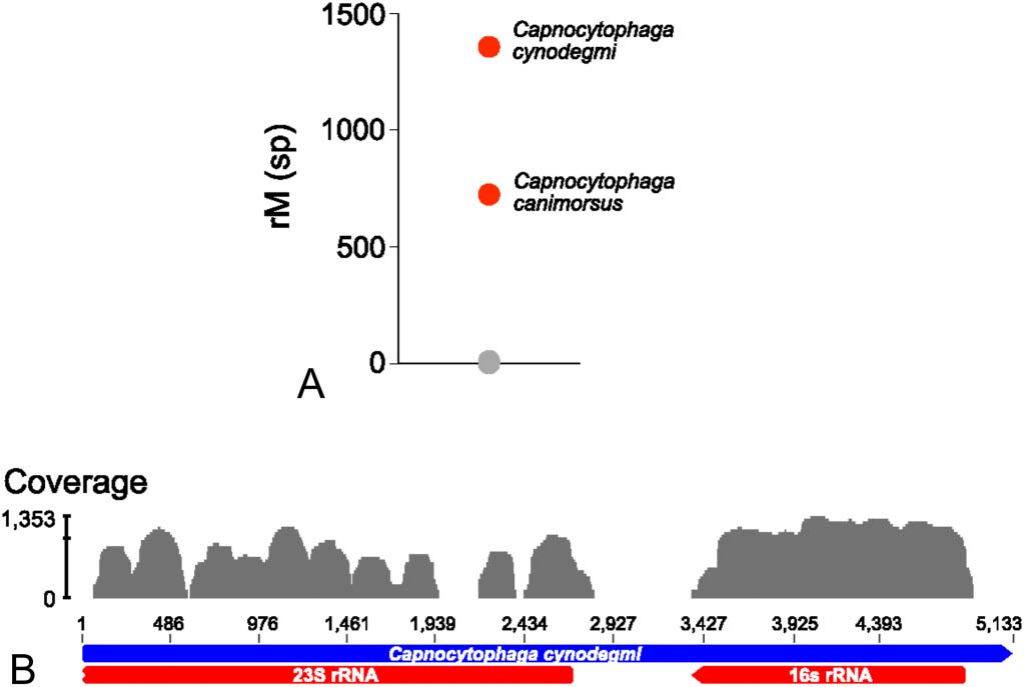
Identification of *Capnocytophaga canimorsus* and *Capnoctyophaga cynodegmi* by metagenomic deep sequencing. A, Organisms identified in the patient's aqueous sample are plotted as a function of matched read pairs per million read pairs (rM) at the species level based on nucleotide alignment. Sequencing reads aligned to *C. canimorsus* and *C. cynodegmi* (red circles) predominated the sample. Gray circles indicate background sequencing reads. B, *Capnocytophaga cynodegmi* sequences from case 2 assembled against the reference *C. cynodegmi* genome (GenBank NZ_CP022378).

A 64-year-old woman with a history of rheumatoid arthritis being treated with rituximab infusions sustained an outdoor foreign body injury after using motorized landscaping equipment. She developed ocular irritation and decreased vision and was treated at an outside facility. She presented 1 month into treatment for consultation after having failed therapy with topical prednisolone acetate 1% and topical ciprofloxacin. Her left cornea disclosed several superior mid-stromal peripheral and tiny paracentral subepithelial infiltrates (Fig. 1B, left). A 1-mm hypopyon was present. Multiple Gram stains, potassium hydroxide (KOH) stains, and cultures obtained from epithelial scrapings over the areas of subepithelial infiltrates were unrevealing. Confocal examination demonstrated nonspecific inflammatory changes. The stromal lesions progressed deeper. Because the scattered superficial infiltrates were clinically concerning for satellite lesions, the patient was treated aggressively with topical, intrastromal, and oral antifungal therapy (including amphotericin B, voriconazole, and natamycin). Over the next 2 months, the patient developed progressive worsening of anterior chamber inflammation associated with endothelial plaques (Fig. 1B, middle). Aqueous fluid from 2 anterior chamber washout procedures as well as corneal punch biopsy and patch graft of the necrotic superior mid-stromal infiltrates (Fig. 1B, right), did not identify any organisms using aerobic and anaerobic media. A robust inflammatory reaction persisted after a patch graft. Aqueous fluid from a third washout procedure was sent to a Clinical Laboratory Improvement Amendments-certified laboratory for universal polymerase chain reaction for fungal genomes and tested negative. Residual aqueous fluid was sent to the Proctor Foundation for metagenomic deep sequencing (MDS). MDS is an unbiased high-throughput sequencing approach that interrogates all potential genomes in a clinical sample. MDS was performed as previously described.^5^ This study adhered to the tenets of the Declaration of Helsinki. The Institutional Review Board of the University of California, San Francisco, approved the study (16-19151), and informed consent was obtained from the patient. Two species of *Capnocytophaga*, *Capnocytophaga canimorsus*, and *C. cynodegmi* were identified (Fig. 2A). Orthogonal validation with partial 16S rRNA gene reverse transcription polymerase chain reaction and Sanger sequencing of the remaining RNA from the patient's aqueous specimen confirmed the presence of *Capnocytophaga* genome (Fig. 2B). The patient was placed on topical clindamycin 5% with subsequent complete resolution of inflammation and infiltration in 6 weeks. After the MDS results, the patient reported that she lives with numerous cats and dogs. Her acuity postresolution is hand motions from irregular astigmatism from the patch graft and dense cataract that progressed during the severe inflammatory episode. Penetrating keratoplasty with cataract surgery is planned.

## DISCUSSION

*Capnocytophaga* is a gram-negative rod-shaped bacterium that is part of the normal oral flora of humans, dogs, and cats.^2,6^ Human infection from *Captocytophaga* is rare. Ocular infection is similarly uncommon, and although endophthalmitis^7^ and blepharoconjunctivitis^8^ are reported, keratitis is the most common clinical presentation. On review of available case reports,^1–4,7^ almost all *Capnocytophaga* ocular infections occur in hosts with secondary risk factors such as immune compromise and trauma. In addition, *Capnocytophaga* keratitis often presents with a fairly rapid course to fulminant inflammation. Deep stromal infiltration with progression to necrosis is common, and infections associated with *Capnocytophaga* are associated with a poor visual outcome.^1–4,7,9^ Although several species of *Capnocytophaga* are part of the normal human oral flora, *C. canimorsus* and *C. cynodegmi* are found only in feline and canine oral flora. A recent report of 7 cases of *Capnocytophaga* keratitis in dogs similarly demonstrates an aggressive course with keratomalacia and poor prognosis.^6^

Herein, we present 2 recent cases of *Capnocytophaga* keratitis, each illustrating some important aspects of this disease. Both cases demonstrated *Capnocytophaga* occurring in the setting of a compromised host. Both cases demonstrated robust ocular inflammation with hypopyon and stromal necrosis. Both cases provided a history of frequent contact with pet dogs and cats. Both cases improved with administration of topical clindamycin.

With case 1, despite prompt identification of *Capnocytophaga*, perforation still occurred within 4 days of presentation. Sensitivity information on unusual pathogens often takes significant time. In vitro *Capnocytophaga* susceptibility testing is further complicated by relatively slow, fastidious, growth of the organism and lack of laboratory standard guidelines for antimicrobial susceptibilities. Review of previous case reports describing the efficacy of topical clindamycin in similar cases was helpful in management of this case.

Case 2 demonstrated the downfall of a ∼60% sensitivity for the current gold standard cornea culture to identify pathogens responsible for infectious keratitis.^10^ Cornea cultures are particularly challenging for deeper infections and infections with an intact epithelium. In addition, with some strains of *Capnocytophaga* being fastidious in culture and difficult to isolate, standard culture techniques may be underestimating its occurrence. In cases in which the suspicion for infection is high but conventional diagnostics are unrevealing, MDS has the potential to provide an actionable diagnosis, as shown with this case. The unbiased nature of MDS, where it can detect any viable pathogen in a clinical specimen, is particularly useful when the causative infection is rare and hence might not be on the differential diagnosis. Although more validation studies are required before MDS can be routinely offered to practicing ophthalmologists, this approach holds promise as a complementary approach to conventional diagnostics for ocular surface or corneal infections.
